# Propagation of Fibrillar Structural Forms in Proteins Stopped by Naturally Occurring Short Polypeptide Chain Fragments

**DOI:** 10.3390/ph10040089

**Published:** 2017-11-16

**Authors:** Irena Roterman, Mateusz Banach, Leszek Konieczny

**Affiliations:** 1 Department of Bioinformatics and Telemedicine, Medical College, Jagiellonian University, 31-530 Krakow, Poland; mateusz.banach@uj.edu.pl; 2 Chair of Medical Biochemistry, Medical College, Jagiellonian University, 31-034 Krakow, Poland; mbkoniec@cyf-kr.edu.pl

**Keywords:** amyloid, hydrophobicity, fibrillation, solenoid

## Abstract

Amyloids characterized by unbounded growth of fibrillar structures cause many pathological processes. Such unbounded propagation is due to the presence of a propagating hydrophobicity field around the fibril’s main axis, preventing its closure (unlike in globular proteins). Interestingly, similar fragments, commonly referred to as solenoids, are present in many naturally occurring proteins, where their propagation is arrested by suitably located “stopper” fragments. In this work, we analyze the distribution of hydrophobicity in solenoids and in their corresponding “stoppers” from the point of view of the fuzzy oil drop model (called FOD in this paper). This model characterizes the unique linear propagation of local hydrophobicity in the solenoid fragment and allows us to pinpoint “stopper” sequences, where local hydrophobicity quite closely resembles conditions encountered in globular proteins. Consequently, such fragments perform their function by mediating entropically advantageous contact with the water environment. We discuss examples of amyloid-like structures in solenoids, with particular attention to “stop” segments present in properly folded proteins found in living organisms.

## 1. Introduction

Protein folding anomalies, particularly in the context of prions [1], cause a range of pathologies, including bovine spongiform encephalopathy (commonly known as mad cow disease) [2] as well as Alzheimer’s disease [3]. This phenomenon has motivated extensive research into protein folding dynamics. Analysis of proteins which fold in an anomalous manner despite retaining correct residue sequences has resulted in assigning the “misfolding disease” moniker to certain pathologies [4]. Such diseases are known to result from causes other than mutations [5]. In other words, a correctly transcribed polypeptide adopts a conformation which favors linear complexation in place of the expected native globular structure. Despite a multitude of studies (including identification of factors which promote amyloidogenesis), the exact mechanism behind this process remains unknown [6]. Published literature is replete with examples of peptides which can be experimentally observed to form amyloids. Interestingly, most of these peptides are dominated by beta folds [7,8]. 

The fuzzy oil drop model, designed to facilitate analysis of protein structures, has emerged as a promising tool in the study of amyloids, and particularly of the conformational changes which enable peptide chains to propagate linearly [9]. The basic assumption behind the model is that a protein can be compared to a spherical (or globular) micelle, where hydrophobicity peaks at the center of the globule and decreases along with distance from the center, becoming negligible on the surface. Any protein can be inscribed in a suitably shaped ellipsoid and the theoretical distribution of hydrophobicity inside such a “capsule” is given by the 3D Gaussian. The ellipsoid itself is defined by its sigma parameters (one for each principal axis). Depending on the relations between these parameters, the resulting micelle can range from spherical (for a perfectly symmetrical protein) through various elongated globules, all the way to cylindrical solenoids and ribbon-like amyloid fibrils [9]. 

Further analysis of this broad spectrum of structural forms leads to a set of proteins containing solenoid fragments. Here, an important problem emerges: how does evolution prevent such fragments from propagating indefinitely? Clearly, unrestricted elongation (or complexation) of solenoids would result in a variety of pathological conditions. It turns out that all protein structures, whether spherical, globular, cylindrical or ribbon-like, can be adequately modeled by a suitable 3D Gaussian, or, more specifically, by adjusting the sigma parameters which determine the shape of the encapsulating ellipsoid. In addition to a mathematical formulation of protein structures, the model also reveals the propagation of hydrophobicity within the protein body. Spherical and globular structures tend to exhibit a single centralized hydrophobicity peak, while in elongated forms (cylinders and ribbons) the distribution is linear, with local peaks propagating along the axis of the micelle. Spherical and globular structures which conform to the 3D Gaussian are easily dispersed and soluble, with deviations from the idealized distribution usually corresponding to sites of biological activity ligand binding [10] and protein complexation [11]. Applying the Gaussian function in the analysis of such structures paves the way towards formalized, quantitative assessment of the scope of deviations between theoretical and observed distributions of hydrophobicity. Notably, any local departure from the idealized Gaussian may also function as a seed for amyloid-like aggregation. Recent research indicates that, with the use of suitable techniques, almost any protein can be induced to transform into an amyloid [12]; thus, it seems that such seeds are ubiquitous in nature.

In principle, any linear structure, especially one which consists of identical peptide chains, requires a “stop” signal to prevent unrestricted propagation. This work analyzes the means by which nature controls the propagation of linear aggregations of hydrophobicity peaks and troughs.

The study set comprises proteins which contain solenoid fragments, i.e., structures where the local hydrophobicity profile can propagate linearly, bracketed by “stop” sequences which prevent excessive propagation.

## 2. Results

The fuzzy oil drop model posits that a stable protein structure can be described by a suitably adapted 3D Gaussian. Solenoids, however, do not meet this condition rather than being centered upon a shared core; they exhibit linear propagation of local hydrophobicity peaks. This propagation requires a “stop” signal to prevent the solenoid from elongating indefinitely. In order to determine which residues comprise the “stop” signal, we focus on fragments directly adjacent to the terminal beta folds which comprise the solenoid. Such fragments have been subjected to analysis based on the fuzzy oil drop model; we expect that knowledge regarding their structure may be reused to design artificial “stoppers” preventing aggregation of amyloid proteins in misfolding diseases.

### 2.1. “Stop” Signals 

Below we provide several detailed examples of proteins which contain solenoids. Additional proteins are mentioned by listing the status of the terminal folds which act as “stop” signals. Table 1 and Table 2 provide an overview of each protein. RD values above 0.5 reveal the absence of a prominent hydrophobic core. Evidently, most of the analyzed proteins diverge from the theoretical hydrophobicity distribution. This is caused by linear propagation of local hydrophobicity patterns in the solenoid fragment, which dominates the structure of each protein.

The status of “stop” fragments themselves varies. In most antifreeze proteins, such fragments are highly accordant with the fuzzy oil drop model. This means that the bracketing fold, which out of necessity is located close to the surface of the protein, does indeed expose its hydrophilic residues outward. In the case of lyases, some “stop” fragments locally deviate from the theoretical model.

**Figure 1:**
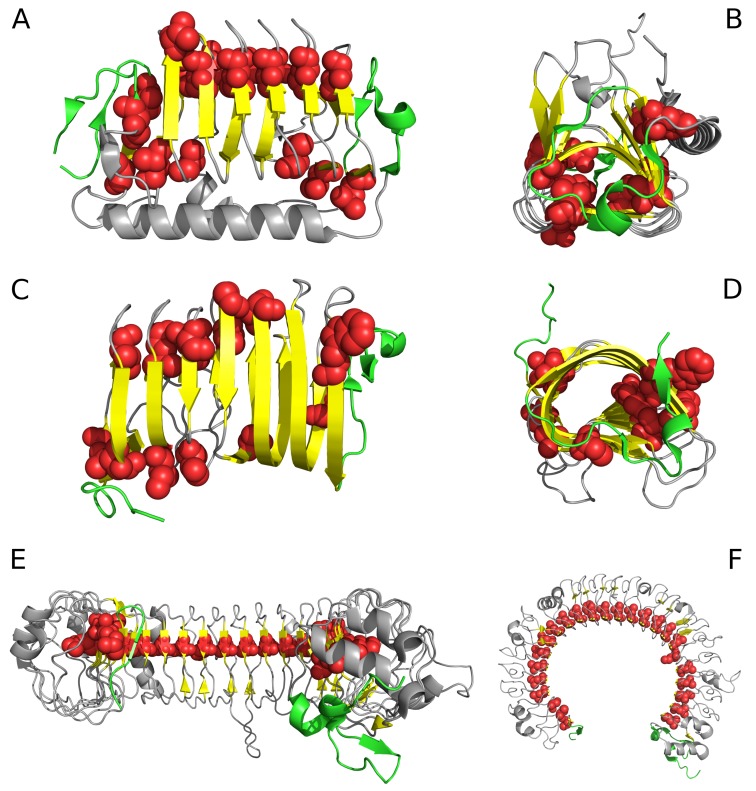
Selected proteins viewed from two different angles. Left column: visualization of linear propagation of hydrophobic residues responsible for local maxima. Right column: visualization of “stop” fragments. (**A**,**B**): 3UYV; (**C**,**D**): 4YZA; (**E**,**F**): 2A0Z. In 2A0Z, the only residue directed toward the center is ILE 510. This protein is not included in our analysis due to its peculiar structural form which does not permit analysis based on the fuzzy oil drop model; however, it is visualized in the figure to show linear propagation of hydrophobic residues. Green fragments: stop fragments. Red space filling presentation: the positions of residues identified by fuzzy oil drop model as highly discordant versus the idealized distribution. This aims to show that they generate the linear propagation accordant to long axis of the solenoid. Gray fragments: additional chain fragments increasing the solubility of protein under consideration. However, this subject is not the object of analysis in this paper. Left column: solenoid axis—parallel to the paper sheet plane (**A**,**C**,**E**), right column—solenoid axis perpendicular to the paper sheet plane (**B**,**D**,**F**).

Analysis of the “stop” fragment in conjunction with the adjacent loop reveals the degree to which the whole system is aligned with the environment and with the structural motif blocked by the “stop” signal. In most cases, good accordance with the FOD model is observed, particularly in the N-terminal section. Proteins - where the “stop” signal is neither at the N- nor at the C-terminal fragment of the chain contain additional long sequences which bind the molecule together, stabilizing it despite the absence of a monocentric hydrophobic core. Examples of structures which exhibit linear propagation of hydrophobicity in their solenoid sections are shown in Figure 1.

**Figure 2:**
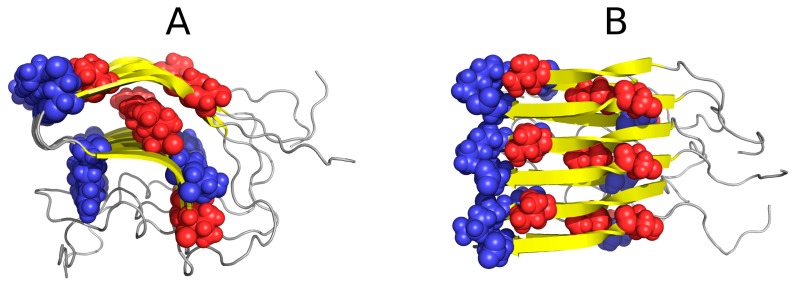
Example of an amyloid fibril (prion protein 2KJ3) in two projections, showing linear distribution of hydrophobicity (red: hydrophobic residues; blue: hydrophilic residues). Since no “stop” signal is present, the structure is susceptible to further linear aggregation. The regularity and of fibril without any polypeptide chain fragment disturbing the linear organization. (**A**,**B**)—different perspective: (**A**)—the fibril long axis—perpendiculat ro the paper sheet, (**B**)—the fibril axis parallel to the paper sheet.

Table 1 and Table 2 also present the status of 2KJ3—a prion protein which is entirely solenoid-shaped and devoid of any “stop” signal. It should come as no surprise that this protein diverges greatly from the theoretical hydrophobicity distribution (in favor of linear propagation, see Figure 2). In Figure 2, it is shown that the linearly propagated high and low hydrophobicity without any fragment interrupting this order may be continued endlessly. 

### 2.2. Lyase—Bacterial Chondroitinase b Pectate Lyase (PDB ID: 1DBG) 

**Figure 3:**
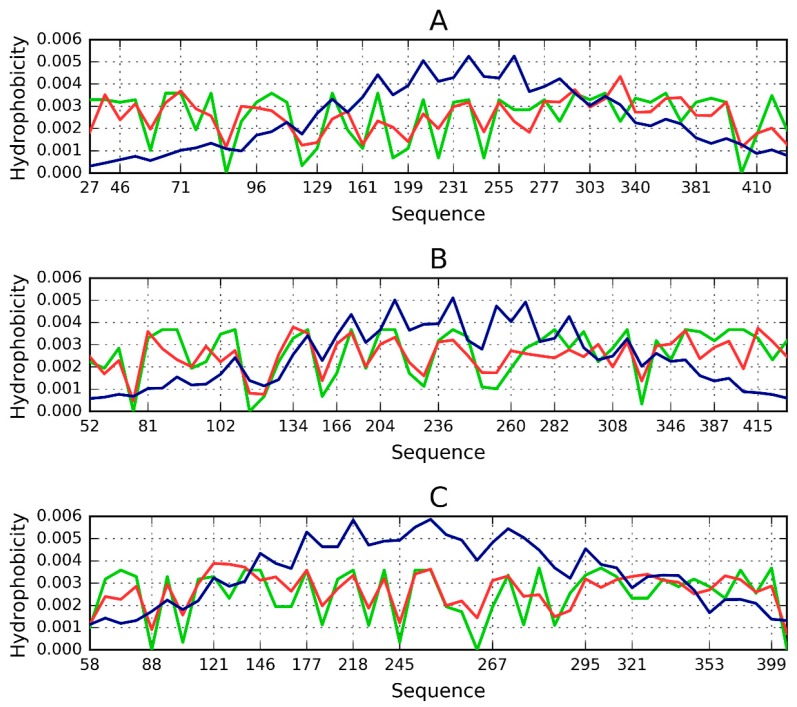
Theoretical (blue), observed (red) and intrinsic (green) hydrophobicity distributions for successive β-sheets forming the lyase solenoid fragment (1DBG). Labels (**A**,**B**,**C**) follow the convention used to identify sheets in PDB. The blue line (T) shows the theoretical concentration of hydrophobicity in the central part, along with low hydrophobicity in the N- and C-terminal section, consistent with the 3D Gaussian. Proteins which exhibit such distribution are water-soluble. In contrast, the red line represents a distribution where no central peak can be observed and where hydrophobicity does not taper off in the terminal section—such as in the case of solenoids. Actual distribution (green) is closely aligned with the red profile, suggesting that the solenoid does not generate a central hydrophobic core and that it is moreover capable of complexing additional hydrophobic molecules in its terminal sections. This phenomenon is thought to be responsible for structural changes leading to formation of fibrillar forms rather than monocentric globules.

**Figure 4:**
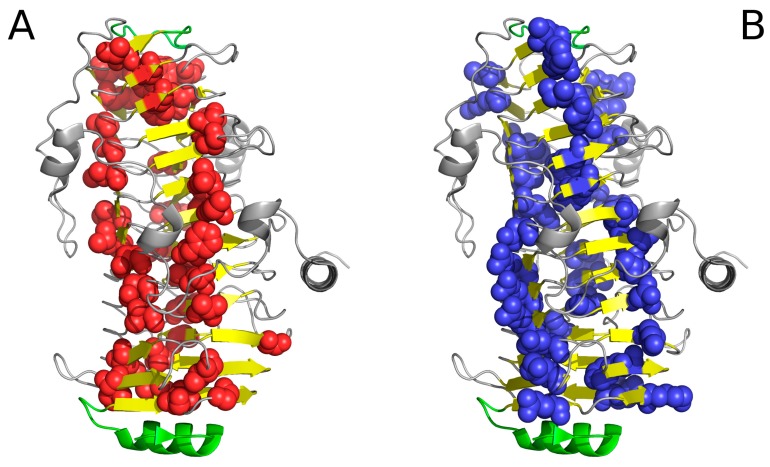
Hydrophobic (**A**) and hydrophilic (**B**) residues in 1DBG. VdW presentation has been applied in both images to highlight selected residues. Note the linear arrangement of hydrophobic and hydrophilic bands. The green helix at the bottom acts as a “stopper”.

As discussed above, an amyloid composed of protein chains where local hydrophobicity peaks propagate linearly can, potentially, grow without limit. Given the existence of proteins which resemble cylindrical micelles, a “stop” signal must be present. To investigate this issue, we analyzed two proteins containing solenoid-like fragments (consistent with our definition of a cylindrical micelle). The study group consists of bacterial chondroitinase b lyase E.C. 4.2.2.19 (1DBG) and Bordetella pertussis virulence factor p. 69—a cell adhesion protein (1DAB). Both proteins are referred to as “mainly β 3 solenoids” in the CATH database (2.160.20.10). Figure 3 shows the structure of the lyase, depicting its linear propagation. The distribution of hydrophobicity in three β-sheets present in 1DBG shown for sequential short β-structural fragments reveals absence of monocentric hydrophobic core (expected maximum in central part). The numbers on the x-axis show the positions of residues present in sequential β-structural fragments. The sinusoidal form of the observed distribution reveals repetitive form of the local maxima followed by local minima of hydrophobicity. It is continued from the beginning till the end of the β-sheet without the lowering neither on N- nor C-terminal fragments. The similar form of the profile of observed and intrinsic hydrophobicity is also very characteristic. The theoretical distribution expresses the expected effect of local organization aimed at hydrophobic core construction. The similarity between observed and intrinsic hydrophobicity suggests the construction of the distribution as result of dominating role of intrinsic hydrophobicity. This linear propagation along the β-sheets propagation parallel to the long axis of the solenoid is shown also on Figure 4 visualizing positions of local maxima and local minima in sequential β-structural fragments. 

Both proteins, taken as a whole, diverge from the theoretical distribution of hydrophobicity. This effect is particularly pronounced in their cylindrical sections. The scope of discordance between the theoretical and observed distribution can be seen in Figure 4, where each beta sheet is analyzed separately. Linear propagation of local hydrophobicity peaks is illustrated in Figure 4. 

**Figure 5:**
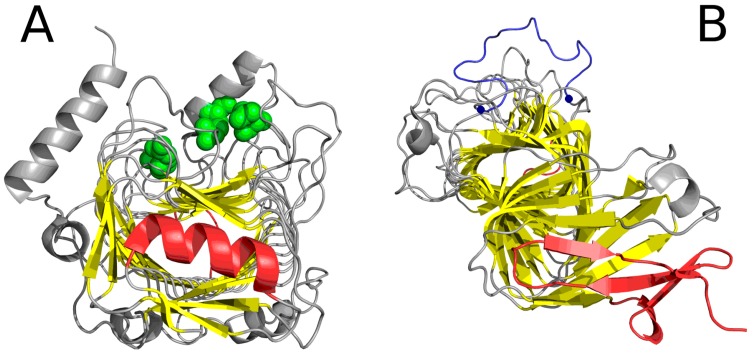
“Stop” signals which accompany solenoids. (**A**) lyase (1DBG): red fragments: helix preventing further propagation of the fibrillar structure; green: residues engaged in enzymatic activity; yellow: β-sheet comprising the solenoid; (**B**) cell adhesion protein (1DAB): red fragments which constitute “stop” signals—an uncoiled loop (front) and a beta fold (red)—preventing propagation of fibrillar forms in either direction; blue: fragment (limited by two blue dots) believed to mediate interaction with epithelial cells [13] (GGXXP)5.

**Figure 6:**
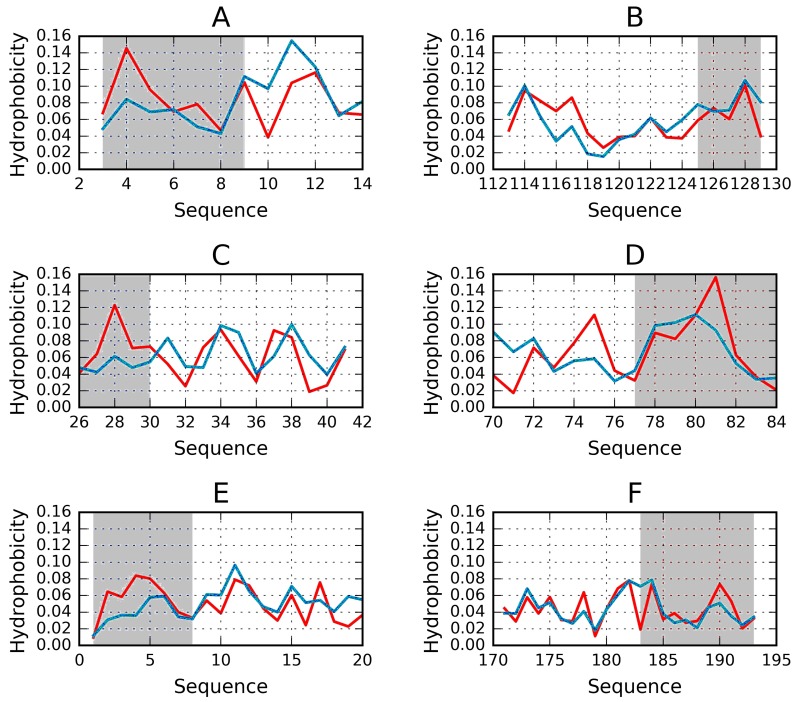
Examples of “stoppers” adjacent to the N- and C-terminal sections of the solenoid. Blue—theoretical distribution, red—observed one. (**A**) N-term 1L0S, (**B**) C-term 3UYV, (**C**) N-term 1DBG, (**D**) C-term 1L1I, (**E**) N-term 1EWW, (**F**) C-term 4YZA.

It is worth noting that both 1DBG and 1DAB also include fragments where the distribution of hydrophobicity is locally consistent with the model. Figure 5 presents the positions of polypeptide fragments where the conformant fragments serve as “stoppers”, preventing amyloid-like propagation of the solenoid. The N-terminal section, located at the center of the cylinder, ensures suitable closure, as does the disordered fragment marked in red. The low values of RD for fragments recognized as stoppers as given in Table 1 and Table 2, together with profiles shown in Figure 6 (particularly Figure 6C) support the treatment of selected polypeptide chains fragments as stoppers. 

Other fragments which also remain locally consistent with the model are marked in green. They are found on the surface of the protein and contribute to its solubility. The cylindrical portion (solenoid) remains highly discordant. Parts of the solenoid (except the outward-facing loops) closely resemble amyloid aggregates, with linearly clustered hydrophobicity peaks and through preventing the protein from adopting a FOD-like conformation.

Analysis of the hydrophobicity distribution in the solenoid fragment indicates linear propagation inconsistent with the fuzzy oil drop model. This is particularly evident in the N- and C-terminal sections, where, contrary to the model, hydrophobicity does not taper off (which would facilitate closure of the ellipsoid). Clearly, both terminal sections are susceptible to further propagation.

Figure 4 reveals a linear arrangement of hydrophobic (A) and hydrophilic (B) bands. Such linear propagation may continue indefinitely in the absence of a “stop” signal. As the protein under consideration is found in healthy organisms, it must be protected against indefinite propagation. The corresponding “stop” fragments are highlighted in Figure 5; they work by disrupting the terminal sections of the solenoid, rendering them consistent with the fuzzy oil drop model (red sections in Figure 5). In particular, the helical fragment shown in Figure 5A is recognized as an amphiphilic helix. This phenomenon may hint upon effective means of arresting linear propagation of fibrillar aggregates in patients suffering from amyloidosis.

The analysis of the importance of the amyloid-like solenoid in lyases focuses on its role as a generator of a specific local force field. Catalytic residues (highlighted in Figure 5A, following [14]) are all adjacent to amyloid-like beta sheets. The reaction catalyzed by lysases requires a nonaqueous environment. Accordingly, the peculiar structure of the protein deviating from the theoretical hydrophobicity distribution may produce a suitable “atypical” local force field, promoting catalysis through cleavage of a single bond in the ligand and formation of a replacement double bond.

1DBG, a lyase bacterial chondroitinase b pectate lyase provides a useful study subject as it contains a helical “stop” signal. This protein was used as a template for the design of other compounds (peptides) which, according to our analysis, may be of use in arresting propagation of linear structures in misfolding diseases [9].

The search for drugs which prevent unchecked aggregation of amyloid fibrils should exploit solutions which have evolved in nature. It turns out that “stop” fragments are uniquely structured to fulfill their biological role. Our work suggests that a potential therapeutic agent should be a helix (4-8 aa) with a highly amphiphilic structure. One of its sides should be strongly hydrophilic, while the opposite side should remain hydrophobic, enabling the helix to interpose itself between the terminal beta fold and the environment, i.e., water. Such conformation would prevent further elongation of the target fibril by making it impossible to recruit another unit chain. Figure 6 illustrates a selection of potential “stopper” signals, appropriate for both the N-terminal and the C-terminal fragment of the solenoid. In each case, we can observe amphipathic distribution of hydrophobicity, the hydrophilic side faces the environment while the hydrophobic side attaches itself to the solenoid.

## 3. Description of Model 

### 3.1. Dataset

We have extracted from the PDB (Protein Data Bank, www.rcsb.org) database a set of proteins whose descriptions include the “solenoid” keyword (Table 3). This set mostly comprises two classes of structures antifreeze proteins and lyases, although we also include cell adhesion proteins, toxins and de novo molecules. Prions are used as a classic example of amyloid proteins.

The proteins included in the above list are all properly folded and derived from their respective source organisms. Comparing the specifics of linear propagation of a hydrophobic force field in amyloids and in solenoids which comprise properly folded proteins provides clues with regard to how unrestricted propagation may be arrested. Solenoids do not propagate indefinitely and do not form multiprotein complexes; this is due to the presence of short fragments which act as “stoppers”. Studying the properties of these fragments may lead to the synthesis of suitable stoppers which would prevent the propagation of amyloid fibrils; note, however, that providing examples of “designer” stoppers matching specific pathological structures goes beyond the scope of this work and will form the subject of a separate publication.

### 3.2. Fuzzy Oil Drop Model

The FOD model has been extensively described in online publications; accordingly, we will limit ourselves to a brief recapitulation of its core concepts relevant from the point of view of the presented study. The model posits the existence of a hydrophobic core which is mathematically modeled by a 3D Gaussian [26,27,28]. The majority of individual domains represent the status of a highly similar distribution of observed hydrophobicity in respect to an idealized one. 

Proteins encountered in living organisms usually exhibit certain localized deviations from this theoretical distribution of hydrophobicity, and these deviations often correspond to sites of biological activity: binding pockets (lower-than-expected hydrophobicity [10]) and complexation sites (excess hydrophobicity on the molecular surface [11]). 

From among the many possible disagreements between theoretical and observed hydrophobicity profiles, one type stands out: the linear propagation of local hydrophobicity peaks/troughs, which are themselves dependent only on the intrinsic hydrophobicity of each residue forming the unit chain, with no centralized (molecule-wide) hydrophobic core. This phenomenon is encountered in amyloid forms [9,27,28]. The fuzzy oil drop model states that the presence of a central core is necessary to ensure the separation of individual molecules and therefore solubility. Even so, we have identified certain proteins in which local hydrophobicity profiles appear to propagate linearly in an amyloid-like fashion. We conclude that such molecules require the presence of a “stop” signal which would prevent indefinite propagation.

A fibril may be generated either by cylindrical or ribbon-like micelles. The former category comprises structures referred to as solenoids, where local hydrophobicity peaks/troughs propagate linearly [9]. Below we provide a description of those aspects of the fuzzy oil drop model which are relevant for the presented study.

The 3D Gaussian (which, according to the model, expresses theoretical hydrophobicity) peaks at the center of the molecule and assumes near-zero values on its surface. Any cross-section of this distribution produces a corresponding 2D Gaussian. This type of distribution is observed in many real-world proteins, where hydrophobic residues congregate at the center of the protein body while hydrophilic residues are exposed on the surface.

Both hydrophobicity distribution profiles—theoretical (*T*: given by the Gaussian) and observed (O: computed by summing up hydrophobic interactions between each residue and its neighbors in a 9Å radius)—can be compared quantitatively. Quantitative expression of the differences between the expected (*T*) and observed (*O*) distribution is enabled by the Kullback–Leibler divergence entropy formula [29]:

(1) $$D_{KL}(p/p^{0})=\sum_{i=1}^{N}\;p_{i}\;\log_{2}(p_{i}/p_{i}^{0})$$

*D_KL_* expresses the distance between the observed (*p*) and target (*p*^0^) distributions, the latter of which is given by the 3D Gaussian (*T*). The observed distribution (*p*) is referred to as *O*.

For the sake of simplicity, we introduce the following notation:

(2) $$O/T=\sum_{i=1}^{N}\;O_{i}\;\log_{2}(O_{i}/T_{i})$$

Given that *D_KL_* is a measure of entropy, it must be compared to a reference value. In order to facilitate meaningful comparisons, we introduce another boundary distribution, referred to as “uniform” or “*R*”, which corresponds to a situation where each effective atom possesses the same observed hydrophobicity (1/*N*, where *N* is the number of residues in the chain). This distribution is deprived of any form of hydrophobicity concentration at any point in the protein body:

(3) $$O/R=\sum_{i=1}^{N}\;O_{i}\;\log_{2}(O_{i}/R_{i})$$

Comparing *O*|*T* and O|R tells us whether the given protein (*O*) more closely approximates the theoretical (*T*) or uniform (*R*) distribution. Proteins for which *O*|*T* > *O*|*R* are regarded as lacking a prominent hydrophobic core. To further simplify matters we introduce the following relative distance (*RD*) criterion:

(4) $$RD=\frac{O/T}{\left(O/T+O/R\right)}$$

**Figure 7:**
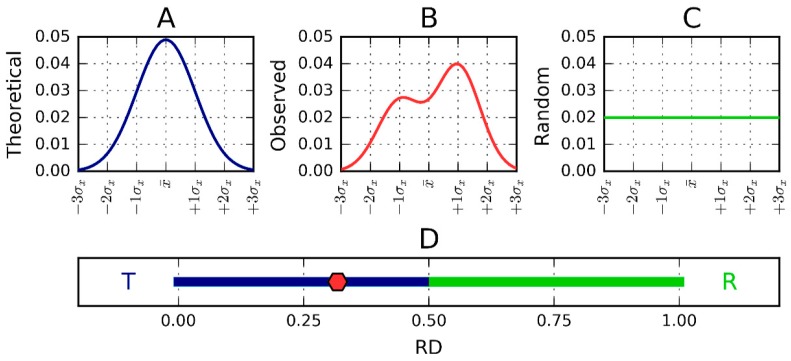
One-dimensional representation of fuzzy oil drop model parameters. The leftmost chart (**A**) presents the idealized Gaussian distribution—*T*—while the chart on the right (**C**) corresponds to the uniform distribution—*R*. Actual hydrophobicity distribution (expressed by the RD parameter) for the target protein is shown in the center (**B**) and marked on the axis with a pink dot (**D**). According to the fuzzy oil drop (FOD) model this protein contains a well-defined hydrophobic core. Vertical axes represent hydrophobicity (in arbitrary units), while horizontal axes represent distance (in multiplicities of σx). According to the three-sigma rule, the range between 0 + 3σ and 0 − 3σ covers more than 99% of the entire probability expressed by the Gaussian. The bottom axis shows the full range of the RD coefficient from 0 (perfect Gaussian) to 1 (uniform distribution with no concentration of hydrophobicity at any point in the protein body). In the presented example, *RD* = 0.318. *RD* > 0.5 is interpreted as a better match for the unified distribution than the theoretical Gaussian, whereas *RD* < 0.5 reveals the presence of a FOD-compliant monocentric hydrophobic core encapsulated in a hydrophilic shell.

This representation is depicted in Figure 7.

Since RD is the relative distance, its value is (according to definition) from the range 0 to 1. *RD* = 0 represents the status of the observed distribution being identical in comparison with the theoretical distribution (Gauss function). *RD* = 1.0 means that the distribution perfectly well represents a unified distribution (no differentiation of hydrophobicity at all). *RD* = 0.5 describes the status of distance versus theoretical (Gauss function) and distance versus unified distribution equal to each other. *RD* < 0.5 (dark blue part of the axis in Figure 7D) means that the distance versus theoretical distribution is lower than versus the unified one. This simply says that the distribution is closer to the theoretical one (as it is shown in Figure 7D, in the dark blue and green part of the axis).

The *RD* parameter can be used to characterize the complete molecule (chain, domain). It can be also applied to identify the status of a selected fragment of a criterion dependent on the object of analysis. It can be the fragment of particular secondary or super-secondary fragment as well, as it is in this paper, with a fragment assumed to play a role of “stopper”. The *RD* parameter calculated for a certain fragment does not require definition of an individual 3D Gauss function. It requires solely the normalization of values *T_i_*, *O_i_* and *R*, for a selected fragment. The *RD* parameter calculated this way characterizes the certain fragment as locally participating (or not participating) in the generation of the monocentric hydrophobic core. It expresses the local order accordant to expected in idealized hydrophobicity distribution. 

### 3.3. Hydrophobicity Scale 

The intrinsic hydrophobicity scale employed in our study is derived from [14], a work which also shows that using a different scale e.g., Kyle, Doolittle [27,30] does not appreciably change the presented model. In theory, any scale can be used. As the distribution of hydrophobicity in the solenoid does not conform to a monocentric core pattern, it is sufficient to plot a reference distribution of hydrophobicity which is equivalent to the intrinsic hydrophobicity of each participating residue.

Evidently, the larger the protein, the less likely it is to contain a single, monocentric hydrophobic core. When a protein lacks such a core, it may be useful to inquire about the reasons behind the observed discrepancies. In the specific cases which are the focus of this work, discordance results from the presence of a solenoid fragment. This type of structure is characterized by linear propagation of local hydrophobicity maxima and minima along the solenoid’s axis, much as in amyloids [9,28]. The fact that linear propagation of a hydrophobicity field occurs both in amyloids and in solenoids, the latter of which are observed in nature, may provide clues regarding possible mechanisms of counteracting unchecked propagation. This is why our work focuses specifically on proteins which include solenoid fragments. Note that *RD* calculations are performed separately for the entire molecule, for the solenoid fragment and for the putative “stop” fragment.

The status of the whole protein (complex, domain) is determined by plotting a 3D Gaussian for the entire structural unit in question. High values of *RD* (especially where *RD* > 0.5, see Figure 7) indicate that no monocentric hydrophobic core is present. In order to determine the causes behind this discrepancy we seek fragments characterized by high *RD*. In the case of our study set, this property is consistently shared by their solenoid fragments. In each case, the solenoid is considered as part of a larger structural unit, i.e., no separate elliptical capsule is constructed for the solenoid itself; instead, *RD* values are computed by assuming a molecule-wide capsule. Similarly, when considering “stop” fragments, we do not construct an independent Gaussian, as such fragments are also analyzed in the context of their parent structural unit. As a result, the ampiphatic helix, which would exhibit high *RD* if analyzed as a separate unit, remains consistent with the theoretical model (low *RD*) when analyzed in the framework of the entire protein; this is because its hydrophobic side faces the interior of the molecule while the opposite (hydrophilic) side is exposed to the water environment. It should be noted that computing *RD* values for a selected fragment of the polypeptide chain requires prior normalization of *T_i_*, *O_i_* and *R_i_* (the aggregate value for the given fragment must be equal to 1.0). Only after such normalization is performed can Kullback-Leibler’s divergence entropy formula be applied. The resulting RD values determine the status of the fragment with regard to the structural unit for which *T_i_*, *O_i_* and *R_i_* were computed.

The goal of the presented analysis is to determine the purpose of “stop” signals in biologically active proteins in hopes that similar “stoppers” could be constructed to arrest unchecked propagation of other fibrillar structures (including amyloids).

### 3.4. Identification of “Stop” Sequences

The presented work constitutes the first step in the search for a generic method of identifying sequences which may prevent propagation of structures characterized by linearized hydrophobicity distribution. Globular proteins, particularly individual domains, represent the distribution similar to idealized one (*RD* < 0.5). Local discrepancy in form of hydrophobicity excess is identified as protein-protein complexation [11]. Local hydrophobicity deficiency is related to ligand binding cavity [10]. Proteins which are classified as discordant in respect to theoretical distribution include proteins with solenoids in their structure as well as amyloids [28]. The problem of amyloids is their unlimited propagation of fibrylar forms. This is due to linear propagation of bands of locally high and low hydrophobicity parallel to the long axis of the fibril. The linear propagation is characterized as impossible to stop by itself without special dedicated fragments of polypeptide chains which play the role of stoppers. Solenoids are present in many naturally occurring proteins without the ability to build the unlimited fibryls. The question addressed to these proteins is: How do they do it? This is why the identification of specific polypeptide chain fragments playing the role of stoppers appeared to be the object of analysis. For this reason, candidate sequences are identified through visual inspection of selected proteins. The resulting set of helical stoppers is then compared to PDBSum data [20]. Since it is difficult to determine whether a well-defined (in the sense of its secondary conformation) helix is sufficient to arrest linear propagation of hydrophobicity, we also take into account adjacent fragments and try to ascertain their contribution to the “stopper” action. Such analysis is particularly useful when the “stop” signal is not a helix but rather an uncoiled loop. In our analysis, sequence ranges are provided separately for solenoids, for “stop” fragments and for adjacent fragments. The prion protein is included in the set of proteins under consideration to show the status of proteins without stop signaling what causes the infinite propagation of fibrilar form of this protein which may appear in form of amyloid.

## 4. Discussion and Conclusions

Peptides performing the role of stoppers in antifreeze proteins which comprise solenoids exhibit RD values lower than 0.5, while the solenoid itself (as well as frequently the entire molecule) typically satisfies *RD* > 0.5. This indicates that even a molecule which lacks a monocentric hydrophobic core may contain fragments whose local hydrophobicity distribution is consistent with the FOD model. Such fragments are found at the termini of the solenoid and create suitable conditions for interaction with water, preventing further propagation or complexation.

A report on our investigation of potential drugs which may prevent propagation of fibrillar forms is the object of other publications. If the evolution selected the helical forms as stoppers for linearly propagated distribution, the same motif may be applied as stoppers for other fibrillar forms of linear propagation, which is observed in amyloids. Both [9] and [30] discuss the subject of amyloidosis in a comprehensive manner and should be treated as a larger whole. The authors therefore recommend that readers familiarize themselves with these papers. A study of the likely mechanisms leading to formation of amyloid fibrils as well as an analysis of possible drug discovery methods is presented in [9]. Treating amyloid fibrils as ribbon-like micelles [9] is proposed as an alternative to the discussion presented in [31].

Given the large variety of solenoids, a specific classification is in order based on such features as the angle of the twist and the handedness of the helix-to-beta relation. The term “beta-arch” has been proposed for certain types of solenoids which resemble structural arches [32,33]. 

Nanotechnology provides examples of synthetic beta-solenoid proteins composed from repeated pentatpeptides [25]. Repetition of structural forms (usually short beta fragments) is intuitively expected given the underlying sequential repetition [34]. Computerized models which predict the emergence of solenoids for given sequences are also available [35]. Induced transformation of solenoids (as they appear in antifreeze proteins) into amyloid fibrils for biomaterial applications is possible via removal of capping structures which would otherwise prevent propagation. This operation results in elongation of fibrillar forms in a controlled way, facilitating production of reinforcing agents for materials such as glue or cement [36]. A study of the characteristics of capping fragments assumed to arrest amyloid fibril formation in amyloidogenesis can be found in [37]. 

Our conclusion is that the fuzzy oil drop model can serve as a valuable tool in identifying solenoids. A criterion based on linear propagation of local hydrophobicity maxima and minima (as shown in [28]) appears useful in this scope.

## Figures and Tables

**Table 1:** RD values calculated for complete structural units, for each “stop” fragment and for each extended “stop” fragment forming the N-terminal part of the solenoid.

PDB ID	RD Protein	N-Terminal				
		RD STOP	STOP Fragment	Fragment Secondary Form	RD STOP + Neighbor	STOP + Neighbor Fragment.
1EWW	0.522	0.297	1–8	loop	0.472	1–20
1L0S	0.527	0.331	3–9	β int	0.520	3–14
1L1I	0.552	0.426	1–13	loop	0.566	1–17
1N4I	0.526	0.461	1–7	loop	0.565	1–14
3UYV	0.610	0.108	68–72	β-structure	0.263	68–81
5B5H	0.676	0.512	48–55	helix	0.542	48–61
2KJ3	0.618	NA	NA	NA	NA	
4OZX	0.627	0.460	23–32	helix	0.484	52–58
4W8Q	0.551	0.544	41–57	β + loop	0.425	30–60
4YFO	0.634	0.481	21–33	helix	0.468	21–38
4YZA	0.591	0.338	−4–10	loop	0.543	−4–25
4Z05	0.600	0.366	−2–9	β-structure	0.628	−2–22
5BP3	0.593	0.353	910–915	β-hairpin	0.465	906–927
5CU1	0.433	0.542	88–94	loop	0.513	82–92
1DBG	0.651	0.414	30–41	helix	0.429	26–45
1DAB	0.743	0.483	1–11	β-structure	0.619	1–22

NA: not applicable.

**Table 2:** Status of solenoid fragments and individual “stop” fragments at the C-terminal end of the solenoid, as well as of extended “stop” fragments which include the adjacent unit fold of the solenoid.

PDB ID	Solenoid		C-Terminal				
	RD	FRAGM.	RD STOP	STOP Fragment	Secondary Form	RD STOP + Neighbor	Neighbor Fragment
1EWW	0.525	9–73	0.372	74–78	loop	0.361	65–81
1L0S	0.478	10–73	0.491	74–81	β int	0.502	72–90
1L1I	0.479	14–76	0.211	77–84	loop	0.386	70–84
1N4I	0.419	8–75	0.464	76–90	loop	0.493	71–90
3UYV	0.622	β-sheets	0.425	125–129	β + loop	0.354	113–129
5B5H	0.594	β-sheets	0.382	99–109	β-hairpin	0.269	101–113
2KJ3	0.618	All residues	NA		NA		
4OZX	0.655	β-sheets	0.275	252–261	β + loop	0.521	200–206
4W8Q	0.551	β-sheets	0.377	255–263	β +loop	0.518	248–263
4YFO	0.634	37–215	0.569	238–253	helix	0.719	223–253
4YZA	0.591	β-sheets	0.479	183–193	β + loop	0.349	171–193
4Z05	0.600	β-sheets	0.493	183–193	helix	0.411	178–193
5BP3	0.593	β-sheets	0.698	1109–1115	helix	0.689	1109–1119
5CU1	0.427	β-sheets	0.408	149–156	β-structure	0.442	149–165
1DBG	0.763	β-sheets	0.145	419–429	loop	0.524	413–435
1DAB	0.780	β-sheets	0.472	517–525	loop	0.376	517–539

NA: not applicable.

**Table 3:** List of proteins subjected to analysis.

PDB ID	Type	Characteristics	Ref.
1EWW	Antifreeze	spruce budworm antifreeze protein at 30 °C	[15]
1L0S	Antifreeze	Choristoneura fumiferana (spruce budworm) antifreeze protein isoform 337	[16]
1L1I	Antifreeze	Choristoneura fumiferana (spruce budworm) antifreeze protein isoform 337	[17]
1N4I	Antifreeze	Solution structure of spruce budworm antifreeze protein at 5 °C	[18]
3UYV	Antifreeze	Solution structure of spruce budworm antifreeze protein at 5 °C	[19]
5B5H	Antifreeze	Hydrophobic ice-binding site conferring hyperactivity on antifreeze protein from a snow mold fungus	[20]
4OZX	Lyase EC4.2.2.3	alginate lyase from Klebsiella pneumoniae	[21]
4YZA	Lyase EC4.2.2.2	Pectate lyase	[22]
4Z05	Lyase EC4.2.2.2	Pectate lyase	[22]
1DBG	LyaseEC4.2.2.19	Chondroitinase B from Pedobacter heparinus	[14]
5CU1	Lyase E.C.4.4.1.3 Dimethylpropiothetin dethiomethylase	dmsp lyase DddQ from Regeria pomeroyi DSS3Dehydrogenase domain	[20]
5BP3	Lyase EC.2.3.1	Dehydratase domain (dh) of a mycocerosic acid synthase-like (mas-like) pks	[23]
2KJ3	Prion	het-s (218–289) prion in its form obtained by solid-state NMR	[24]
4W8Q	Toxin	truncated hemolysin A from P. Mirabilis	[20]
4YFO	De novo protein	Beta1_ex1	[25]
1DAB	Cell adhesion	P.69 pertactin from Bordetella pertussis	[13]
